# RANK-Dependent Autosomal Recessive Osteopetrosis: Characterization of Five New Cases With Novel Mutations

**DOI:** 10.1002/jbmr.559

**Published:** 2011-11-09

**Authors:** Alessandra Pangrazio, Barbara Cassani, Matteo M Guerrini, Julie C Crockett, Veronica Marrella, Luca Zammataro, Dario Strina, Ansgar Schulz, Claire Schlack, Uwe Kornak, David J Mellis, Angela Duthie, Miep H Helfrich, Anne Durandy, Despina Moshous, Ashok Vellodi, Robert Chiesa, Paul Veys, Nadia Lo Iacono, Paolo Vezzoni, Alain Fischer, Anna Villa, Cristina Sobacchi

**Affiliations:** 1 Institute of Genetic and Biomedical Research (IRGB), Milan Unit, National Research CouncilMilan, Italy; 2 Istituto Clinico Humanitas IRCCSRozzano, Italy; 3 Fondazione Humanitas per la RicercaRozzano, Italy; 4 University of Aberdeen, Musculoskeletal Research Programme, Institute of Medical SciencesAberdeen, United Kingdom; 5 University Children's HospitalUlm, Germany; 6 Institute of Medical Genetics and Human GeneticsBerlin, Germany; 7 Max Planck Institute for Molecular GeneticsBerlin, Germany; 8 INSERM U768, Hôpital Necker-Enfants MaladesParis, France; 9 Université René Descartes, Faculté de Médecine René DescartesParis, France; 10 Unité d'Immunologie et d'Hématologie Pédiatriques, Hôpital Necker-Enfants MaladesParis, France; 11 Great Ormond Street Children's HospitalLondon, United Kingdom

**Keywords:** OSTEOPETROSIS, RANK, IMMUNE SYSTEM, TRANSPLANTATION, HYPERCALCEMIA

## Abstract

Autosomal recessive osteopetrosis (ARO) is a genetically heterogeneous disorder attributed to reduced bone resorption by osteoclasts. Most human AROs are classified as osteoclast rich, but recently two subsets of osteoclast-poor ARO have been recognized as caused by defects in either *TNFSF11* or *TNFRSF11A* genes, coding the RANKL and RANK proteins, respectively. The RANKL/RANK axis drives osteoclast differentiation and also plays a role in the immune system. In fact, we have recently reported that mutations in the *TNFRSF11A* gene lead to osteoclast-poor osteopetrosis associated with hypogammaglobulinemia. Here we present the characterization of five additional unpublished patients from four unrelated families in which we found five novel mutations in the *TNFRSF11A* gene, including two missense and two nonsense mutations and a single-nucleotide insertion. Immunological investigation in three of them showed that the previously described defect in the B cell compartment was present only in some patients and that its severity seemed to increase with age and the progression of the disease. HSCT performed in all five patients almost completely cured the disease even when carried out in late infancy. Hypercalcemia was the most important posttransplant complication. Overall, our results further underline the heterogeneity of human ARO also deriving from the interplay between bone and the immune system, and highlight the prognostic and therapeutic implications of the molecular diagnosis. © 2012 American Society for Bone and Mineral Research

## Introduction

Autosomal recessive osteopetrosis (ARO) is a rare genetic bone disease characterized by increased bone density because of failure in bone resorption. Two main forms can be distinguished on the basis of the presence or absence of osteoclasts, as assessed through bone biopsy, when available.^1^, ^2^ In the osteoclast-rich form, which comprises the large majority of ARO cases, a normal to high number of mature, nonfunctional osteoclasts is present, whereas in the osteoclast-poor form these specialized cells are absent because of a defect in osteoclast differentiation. Two genes have been involved so far in the pathogenesis of this latter form in humans, namely, *TNFSF11 (RANKL*) and *TNFRSF11A (RANK)*.^3^, ^4^ In bone, the *TNFSF11* gene encodes the main osteoclast differentiation factor produced by osteoblasts and stromal cells, while its receptor, RANK, is a transmembrane protein expressed on the surface of preosteoclasts and mature osteoclasts.^5^ Therefore, the osteoclast defect is cell autonomous in the case of *TNFRSF11A* mutations, but is noncell autonomous when RANKL production is defective. This diverse pathogenesis explains the differing behavior of the two subsets of osteoclast-poor ARO patients; osteoclast precursors derived from RANKL-ARO patients are able to differentiate in vitro after exposure to M-CSF and RANKL, but the patients do not respond to hematopoietic stem cell transplantation (HSCT) in vivo, whereas for the RANK-ARO patients, the opposite is true.^2^–^4^

Interestingly, the RANK receptor can activate several signaling pathways, which are functional not only in the osteoclast lineage but in other tissues as well, including immune cells, as shown by the immunological phenotype displayed by both *Tnfsf11*^*−/−*^ and *Tnfrsf11a*^*−/−*^ mice dominated by absence of lymph nodes.^6^–^10^ However, no major immunological defects have been identified in RANKL-deficient patients,^3^ whereas a partial defect in peripheral B cell maturation, sometimes associated with a mild hypogammaglobulinemia, was reported in RANK-deficient patients by our group.^4^ Nevertheless, the results of immunological investigations previously performed on RANK-dependent ARO should be regarded as preliminary, because of the difficulty in obtaining adequate material from patients affected by this very rare pathology.

Because the description of RANK-ARO patients is limited to the original report,^4^ these issues need further analysis. We report here the identification of five previously unpublished RANK-dependent ARO patients bearing a total of five novel mutations. A detailed characterization of their clinical history showed an increasing heterogeneity in this rare subgroup of ARO patients.

## Materials and Methods

### Mutation analysis

Specimens, including blood and DNA samples, were collected from patients after their parents provided informed consent. Clinical, radiological, and laboratory data were collected for genotype–phenotype correlation studies. This research complies with the standards established by the local Ethical Committee and the granting agency.

Sequence analysis of the *TNFRSF11A* gene (transcript ID number NM_003839) was performed as previously described.^4^ In the case of new missense mutations, at least 100 chromosomes from normal unrelated donors from the same geographical area were also investigated by direct sequence analysis.

### In vitro differentiation of human osteoclasts and confocal microscope analysis

Human osteoclasts were generated by culture of peripheral blood monocytes with M-CSF and RANKL using a standard protocol. Peripheral blood mononuclear cells (PBMCs) were isolated from heparinized blood samples by Ficoll density gradient centrifugation (Biochrom, Cambridge, UK). PBMCs were cultured either on glass coverslips for differentiation analysis or on dentine discs for resorption assays in alpha MEM (Lonza, Walkersville, MD, USA), 10% FCS (Gibco, Grand Island, NY, USA), 1% Ultraglutamine (Lonza), 1% Pen/Strep, 25 ng/mL human M-CSF (R&D, Minnespolis, MN, USA), and 30 ng/ml Rankl (Peprotech, Rocky Hill, NJ, USA). Cells were cultured for 2 weeks with medium changes twice weekly. Cells on coverslips were fixed in 4% paraformaldehyde (PFA) in 1× phosphate-buffered saline (PBS) and then stained with Phalloidin-Alexa 488 (Molecular Probes, Eugene, UT, USA), DAPI (Molecular Probes), and tartrate-resistant acid phosphatase (TRAP) activity using Naphtol-AS-MX-Phosphate (Sigma, St. Louis, MO, USA) and Fast-Red-Violet LB (Sigma). TRAP-positive cells with ≥3 nuclei and actin rings were counted as osteoclasts. Dentine discs were cleaned with 1% SDS and resorption pits were visualized by black ink.

### Expression analysis

Osteoclasts were lysed in Trizol (Invitrogen, Carlsbad, CA, USA) at day 14 of culture. After mRNA isolation according to the protocol provided by the manufacturer, cDNA synthesis was performed using the Revert Aid kit (Fermentas, Hanover, MD, USA) and random hexamers. Quantitative PCR was performed using the Taqman 7500 Real-Time PCR System (Applied Biosystems, Bedford, MA, USA) and the SYBR Green reagent (Applied Biosystems). *TNFRSF11A*- and *GAPDH*-specific primers were used for target gene and endogenous control amplification, respectively. Primer sequences are available on request. RQ values were calculated by the SDS software (Applied Biosystems).

### Enzyme-linked immunosorbent spot

Plasma cells secreting IgG, IgM, or IgA were detected using an enzyme-linked immunosorbent spot assay. Briefly, 96-well plates (Millipore, Bedford, MA, USA) were coated with 10 µg/mL purified goat antihuman IgG, IgA, IgM (SouthernBiotech, Birmingham, AL, USA). After washing and blocking with PBS containing 1% (w/v) bovine serum albumin for 30 minutes, serial dilutions of peripheral blood (PB) or bone marrow (BM) mononuclear cells were added and incubated overnight at 37°C. Plates were then washed and incubated with isotype-specific secondary antibodies, followed by streptavidin–horseradish peroxidase (Sigma-Aldrich, St. Louis, MO, USA). The assay was developed with 3-amino-9-ethylcarbazole (Sigma-Aldrich) as a chromogenic substrate.

### Flow-cytometry analysis

For patients 8A and 8B the immunological characterization was carried out at Hôpital Necker-Enfants Malades (Paris, France). Peripheral blood mononuclear cells were stained with APC anti-CD19, FITC anti-CD27, PE anti-IgD (all from BD Biosciences Pharmingen, San Diego, CA, USA) and Biotin anti-IgM (Jackson ImmunoResearch, West Grove, PA, USA). Percentages of CD27^+^ and switched (CD19^+^/IgD^−^/CD27^+^) B cells were determined by gating on CD19^+^ and CD19^+^/CD27^+^ B cells, respectively. The analysis was performed with FACScalibur (Becton Dickinson, Fullerton, CA, USA).

For patient 9 the immunological characterization was carried out at the Istituto Clinico Humanitas (Milan, Italy). Mononuclear cells from the PB and BM of the patient were purified by standard density gradient technique and labeled with the following antibodies: Pe-Cy7 anti-CD19, FITC anti-CD24, PE anti-CD38, APC anti-CD21, APC anti-CD27, FITC anti-IgM, FITC anti-IgD (BD Biosciences Pharmingen). Samples were acquired on a FACSCanto II system (BD Pharmingen) and analyzed with FLOWJO software (version 4.5.4; Tree Star Inc., Ashland, OR, USA).

## Results

### Genetic findings

**Fig. 1:**
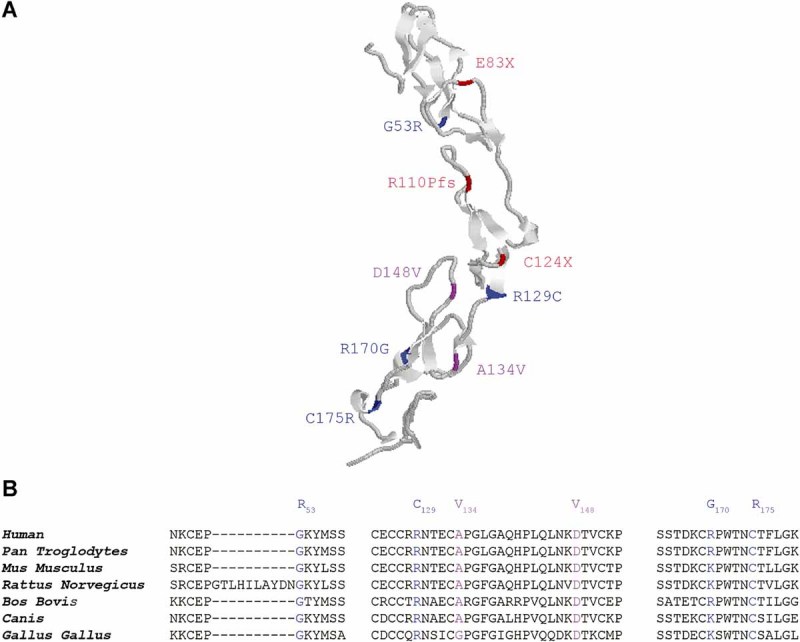
Mutations in relation to the RANK protein structure. (*A*) Three-dimensional model of extracellular domain of human RANK protein. The mutations found to date, in this domain, in our patients are depicted as follows: mutations previously published^4^ are in blue, novel missense mutations are in magenta, novel nonsense and frameshift in red. Human RANK three-dimensional protein structure was determined by homology modeling using the protein structure of mouse rank (downloadable from http://www.rcsb.org:3ME4.pdb) as a template. Modeling was performed by means of SWISS-MODEL tools (http://swissmodel.expasy.org/) on the basis of the CLUSTALW alignment between human RANK primary sequence as target protein, (NP003830.1) and the 3ME4 mouse rank template. Model quality assessment tools were used to estimate the reliability of the resulting model. (*B*) Alignment of RANK protein sequences from several species in the regions covering the missense mutations identified. The mutated residues are indicated above the sequences. The corresponding residues in the individual sequences are colored, missense mutations previously published in blue, novel missense mutations in magenta. The graphical view of domain structure was obtained by ProtoNet (automatic hierarchical classification of protein sequences).

Five new RANK-dependent ARO patients (see next section for the clinical findings) were screened for genes responsible for human ARO. This led to the identification of biallelic mutations in the *TNFRSF11A* gene in each patient, for a total of five novel mutations (shown in red and magenta in Fig. 1): two missense, two nonsense, and a single-nucleotide insertion.

Patient 8A was homozygous for a c.443A > T mutation causing a p.Asp148Val amino acid change and his consanguineous parents were heterozygous for the same nucleotide change. Subsequently we analyzed his affected double first cousin (patient 8B; the family pedigree is shown in Supplementary Fig. 1) and, as expected, found the same mutation at the homozygous state.

Patient 9 was homozygous for the single nucleotide insertion c.328_9insC leading to frameshift and premature termination (p.Arg110ProfsX52). The same mutation was found in the heterozygous state in her consanguineous parents.

Patient 10 was homozygous for a transition (c.401C > T) leading to a novel missense mutation (p.Ala134Val); both her consanguineous parents were heterozygous for this nucleotide change.

Patient 11 was a compound heterozygote for two transversions, c.247G > T and c.372C > A, causing a stop at codon 83 (p.Glu83X) and 124 (p.Cys124X), respectively. His father was heterozygous for the first mutation and his mother for the second one.

The missense substitutions (p.Asp148Val and p.Ala134Val) were not found in more than 100 chromosomes from healthy unrelated individuals from the same geographical areas and therefore are unlikely to be neutral polymorphisms. Alignment of RANK protein sequences from several species further supports this idea, because the mutated residues are strongly conserved in evolution (Fig. 1*B*). Both amino acid substitutions fall in the extracellular domain of the receptor and are likely to affect the binding to the ligand. The two nonsense mutations and the insertion are predicted to lead to the production of N-terminally truncated forms of RANK protein, lacking the entire transmembrane and intracellular domains.

### Clinical evaluation of patients

We reviewed the clinical history of these new RANK-dependent patients in order to better define the features of this subset of ARO (Table 1).

**Table 1:** Clinical and Laboratory Features

Clinical data	Pt 8A	Pt 8B	Pt 9	Pt 10	Pt 11
Age at onset	1 month	At birth	At birth	At birth	2 months
Bone fractures	multiple	no	multiple	one	no
Neurological defects	Blindness (10 mo)	No visual impairment (5 mo)	Congenital blindness	Blindness (22 mo)	
				Hydrocephalus	Blindness (2 mo)
				Additional multiple defects	Hydrocephalus
				Chiari type 1 malformation	
Hepatosplenomegaly	important	no	mild	no	mild
Other features	Gastrooesophageal reflux	Asymptomatic hypocalcemia	Huge skull deformation	Neonatal hypocalcaemia and respiratory acidosis	Gastrooesophageal reflux
				Gastrooesophageal reflux	Lactose intolerance
Hb (g/dL)	9.1	8.7	10.7	8.4	9
Plt (×10^9^/L)	259	283	155	195	290
IgG (mg/dL)	197 (335–623)	261 (235–437)	1140 (470–1550)	309 (240–880)	279 (240–880)
IgM (mg/dL)	49 (48–136)	86 (34–95)	121 (40–280)	20 (10–50)	18 (10–50)
IgA (mg/dL)	<6 (27–86)	7 (2–62)	86 (21–321)	37 (20–100)	38 (20–100)
Age at HSCT	21 months	5 months	12 years	3 years	11 months
HSC origin	Peripheral blood	Bone marrow	Bone marrow	Bone marrow	Bone marrow
Donor	MUD	MRD	MRD	MUD	MRD
T cell depletion	no T-cell depletion	no T-cell depletion	no T-cell depletion	T-cell depletion	no T-cell depletion
Engraftment (% donor chimerism)	Yes (100% donor)	Yes (95% donor)	Yes (100% donor)	Yes (100% donor)	Yes (100% donor)
Bone remodeling	Improved	Ongoing	Improved	Ongoing	Improved
Outcome	Alive and well 14 months post-HSCT	Alive and well 3.5 months post-HSCT	Alive and well 15 months post-HSCT	Alive and well 4 months post-HSCT	Alive and well 3 years post-HSCT
Follow-up	Severe hypercalcemia	Moderate hypercalcemia	Severe hypercalcemia	Severe hypercalcemia	Mild hypercalcemia
	Left tibia fracture	No fractures	Bone fractures	Nephrocalcinosis	No fractures
	Nephrocalcinosis	Acute respiratory distress syndrome	Nephrocalcinosis, GvHD	Respiratory distress	Eosinophilc enterocolitis

Ig levels reported in brackets are normal values for age-matched healthy controls.

All the laboratory data reported refer to the time of the first diagnosis, with the exception of Pt 9.

MUD: matched unrelated donor.

MRD: matched related donor.

Patient 8A is the first child of consanguineous Kurdish parents (Supplementary Fig. 1). He was treated at 1 month of age for severe gastrooesophageal reflux. At the age of 6 months, he presented with noisy breathing. X-rays documented increased bone density of the skull base, diffuse osteosclerosis, and multiple signs of previously unrecognized fractures in X-rays of the complete skeleton consistent with the diagnosis of osteopetrosis. At that time, no hematological anomalies or hypocalcemia were noted. Furthermore, visual evoked potentials (VEPs) were reported to be completely normal at the age of 7 months, so that no indication for hematopoietic stem cell transplantation (HSCT) was thought to be present. However, from the age of 8 months, rapid deterioration of the vision was observed, leading to almost complete blindness at the age of 10 months as documented by the absence of VEPs. A magnetic resonance imaging (MRI) scan at 15 months of age confirmed bilateral narrowing of optic nerve foramina with important nerve compression. Therefore, the decision to perform HSCT was taken (see HSCT and Follow-up), supported by the result of the genetic analysis showing the presence of a homozygous mutation in the *TNFRSF11A* gene. Interestingly, this patient did not show important hematological impairment besides a moderate anemia (at 21 months of age, before HSCT, hemoglobin was 8.6 g/dL with a mean corpuscular volume of 63 fl), while he developed important splenomegaly (122 mm), hepatomegaly (95 mm), and mild thrombocytopenia (113 × 10^9^ platelets/L).

His double first cousin, patient 8B, was investigated soon after birth because of the positive family history. He presented with asymptomatic hypocalcemia at birth (total calcium 1.52 mmol/L with 0.80 mmol/L ionized calcium); subsequent radiological investigations showed diffuse sclerosis of skull and spine and abnormal bone modeling of long bone metaphyses. Genetic analysis confirmed the presence of the same homozygous mutation found in his cousin. At 5 months of age he received HSCT (see HSCT and Follow-up). No visual impairment was documented and only a moderate anemia was reported (Hb 8.7 g/dL, 75 × 10^9^ reticulocytes/L, 283 × 10^9^ platelets/L, 10.2 × 10^9^ leucocytes/L, 1.4 × 10^9^ neutrophils/L, before conditioning for HSCT). A careful immunological characterization of patients 8A and 8B was carried out before transplantation (see Immunological Investigations).

Patient 9 is the first child of consanguineous parents from the Black Forest, Germany. She was referred to us for molecular investigation when she was already 10 and was described as an unusual case of osteopetrosis presenting a severe bone phenotype with important jaw and skull deformations and multiple fractures, congenital blindness, but nearly no impairment of hematopoiesis. The bone biopsy showed highly mineralized bone tissue and irregular net-like trabecular structure. Intertrabecular spaces were scarce and showed neither adipocytes nor islands of hematopoiesis. Osteoclasts were not detected using conventional staining methods (Supplementary Fig. 2). At the age of 12 years, the clinical status of the patient worsened, with the appearance of hematological symptoms, osteomyelitis of the maxilla, and deep apnoea phases because of deformities of the jaw bones requiring tracheostomy. Therefore, supported by the result of the mutational analysis, HSCT was performed. As in patients 8A and 8B, immunological investigations were carried out before transplantation (see Immunological Investigations).

Patient 10 is the first child of consanguineous parents from Pakistan. She was investigated from the neonatal period onwards because of hypocalcemia, poor feeding, hypotonia, subclinical seizures, and respiratory acidosis. She was noted to have a significant gastrooesophageal reflux. She later developed a bulbar palsy (confirmed on EMG) and a right intraventricular hemorrhage. Skeletal X-rays revealed a generalized increase in bone density with loss of corticomedullary differentiation and abnormal appearance of metaphyses, in addition to a minimally displaced fracture of the distal left ulna. She had two episodes of right-sided weakness, considered to be of vascular origin but no abnormality was found on an MRI in the left hemisphere. When she was 19 months old, her vision was noted to be deteriorating and an MRI scan showed increased intracranial pressure; she therefore underwent ventriculostomy at 23 months. After a transient improvement, her vision worsened again and she had a VP shunt inserted. This significantly improved her feeding problems but did not restore vision and flash VEPs have shown only background noise ever since. Sleep studies, performed when she was 24 months old, showed severe obstructive sleep apnoea. Despite the very early onset of the bone phenotype, the child developed only moderate anemia and no hepatosplenomegaly.

Patient 11 was referred to us for molecular diagnosis after HSCT was performed at 11 months of age. Overall, compared with the other patients here reported, he had a more classical clinical presentation. He was the second child of unrelated parents. At the age of 2 months he was noted to be macrocephalic and there were also concerns regarding his vision. An MRI scan showed hydrocephalus, requiring the insertion of a VP shunt, and bilateral optic atrophy associated with complete visual loss. He was also found to have moderate hepatosplenomegaly and anemia. A skeletal survey showed appearance of bone typical of ARO but without obvious signs of fractures. The child had also a number of gastrointestinal problems including gastrooesophageal reflux, lactose intolerance, and failure to thrive.

### Osteoclast differentiation and function in patient 9

Cells for the osteoclast differentiation assay were available only for patient 9. Because she had not yet been transplanted at 10 years of age, we were able to obtain several samples and the assay was carried out in parallel in different laboratories. Of note, the analysis of in vitro osteoclast differentiation was double blind with respect to the result of the molecular analysis of this patient. *TNFRSF11A* mutations previously identified in ARO patients correlated, as expected, with the inability to differentiate osteoclast precursors in vitro after exposure to M-CSF/RANKL, because the defect is cell-autonomous.^4^, ^11^

**Fig. 2:**
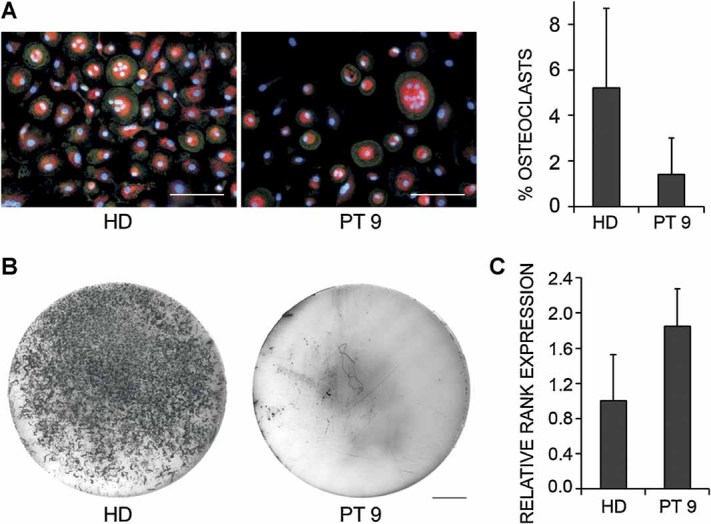
Osteoclast differentiation and function for patient 9. (*A*) Fluorescence images of osteoclasts cultured on glass coverslips, generated from PBMCs of patient 9 and one healthy donor (HD), in concurrent cultures in the presence of M-CSF and RANKL. Osteoclasts were stained for tartrate-resistant acid phosphatase (TRAP) activity (*red*); actin (*green*); nuclei were stained with DAPI (*blue*). Scale bar = 100 µm. Bar graph reports the percentage of TRAP-positive multinucleated (more than three nuclei per cell) cells in patient 9 and in the healthy donor. Results are the average of three cultures. The values are not significantly different. (*B*) Resorption assay for osteoclasts from patient 9 and healthy donor cultured on dentine disc for 14 days. Pits were visualized by ink staining. Representative discs are shown. HD cells showed resorption in five of six dentine discs whereas no pits were detected in all six of patient 9 dentine discs. Scale bar = 1 mm. (*C*) Quantitative RT-PCR of *TNFRSF11A* mRNA expression in cultured osteoclasts. No reduced expression of RANK could be detected in patient 9 cells compared with HD. Results are the average of three cultures.

Unexpectedly, at variance with these results, in vitro osteoclast differentiation from patient 9 showed the formation of a number of TRAP-positive multinucleated cells in the presence of macrophage-colony stimulating factor (M-CSF) and RANKL (Fig. 2*A*). These cells, however, failed to resorb bone, when cultured on dentin (Fig. 2*B*), in agreement with the osteopetrotic phenotype of this patient.

The possibility that the RANKL/RANK pathway could maintain partial activity in this patient was considered. Quantitative reverse transcription-polymerase chain reaction (RT-PCR) revealed that, despite the early stop codon induced by the frameshift mutation, the mRNA isolated from cultured osteoclasts was not subjected to nonsense-mediated decay (Fig. 2*C*). To further verify this hypothesis, cultures of M-CSF-dependent macrophages derived from patient 9 and a healthy donor were stimulated with RANKL or tumor necrosis factor (TNF)-α (as a control) for up to 20 minutes and analyzed for NFκB-p65 translocation. The cells responded to treatment with TNF-α as expected. When treated with RANKL, the patient cells did show a time-dependent increase in the percentage of cells with nuclear p65, although not as pronounced as in cultures of cells from the healthy donor (data not shown). These data suggested that partial RANKL-dependent NFκB activation in cells from this patient was present in vitro, although these experiments could not be confirmed because of lack of available material from patient 9, who had undergone transplantation in the meantime.

### Immunological investigations

Because genetic defects in the *TNFRSF11A* gene have been previously linked to an impairment in peripheral B cell maturation in some patients,^4^ in three new RANK-dependent patients whose pre-HSCT blood cells were available we performed an immunological characterization of the B cell compartment using markers CD19, IgD, and CD27. In this way three subpopulations can be identified: naïve B cells, defined as CD19^+^/IgD^+^/CD27^−^, representing the bulk of the circulating and resident follicular B cells that have never been exposed to antigens; memory B cells (CD19^+^/IgD^+^/CD27^+^), representing a primitive low affinity type of cell; switched memory B cells (CD19^+^/IgD^−^/CD27^+^), which are the effectors of the high affinity adaptive response, giving rise to the long-living plasma cells.

**Fig. 3:**
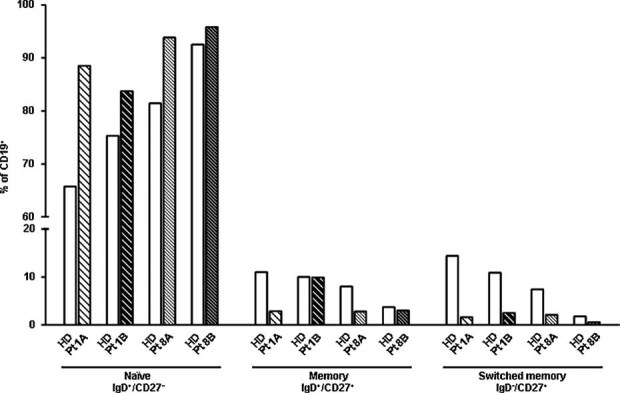
Analysis of B cell compartment in patients 8A and 8B. Percentage of naive (IgD^+^/CD27^−^), memory (IgD^+^/CD27^+^) and switched memory B cells (IgD^−^/CD27^+^) in patients 8A and 8B (21 and 5 months old at analysis, respectively) herein described in comparison with patients 1A and 1B (6 and 3 years old at analysis, respectively) previously described by our group.^4^ As a control, the percentages of healthy donors (HD groups) of the corresponding age group^12^ for each patient are reported.

In agreement with our previous findings, in the two cousins here described, patient 8A and 8B, the cytofluorimetric analysis revealed a reduction in switched memory B cells (IgD^−^/CD27^+^) in the periphery even though less pronounced than in the two siblings reported before (Fig. 3). The milder defect in patients 8A and 8B could be because of their younger age as compared with patients 1A and 1B;^4^ in fact, the percentage of switched memory B cells is known to increase with age.^12^

**Fig. 4:**
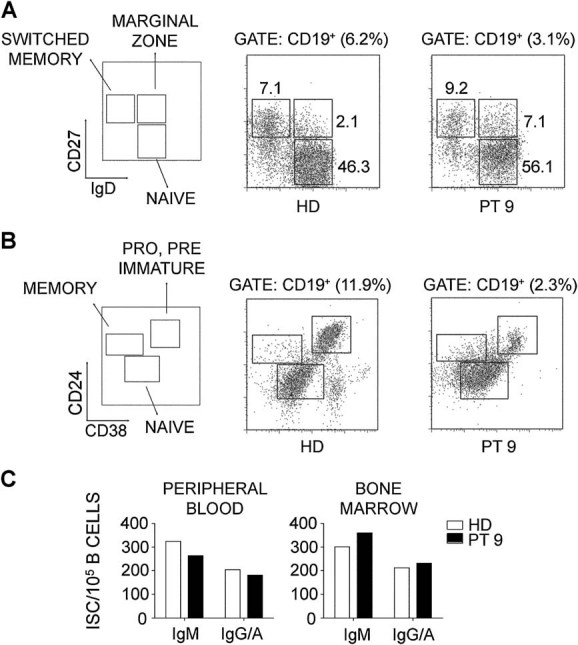
Analysis of B cell compartment in patient 9. (*A*) Peripheral blood mononuclear cells from patient 9 and a representative age-matched healthy donor (HD) were stained with anti-CD19, -CD27, and IgD mAbs and analyzed by flow cytometry. FACS plots are gated on CD19^+^ cells. Numbers indicate percentage of cells for each subset. (*B*) BM-derived mononuclear cells from patient 9 and a healthy donor were stained with anti-CD19, -CD24, and -CD38 mAbs. FACS plots shown are gated on CD19^+^ cells. Numbers indicate percentage of cells for each subset. (*C*) Frequency of Ig-secreting cells (IgM and IgG-IgA isotypes) in cultures for patient 9 (black) and from a healthy donor (*white*). The number of spots/10^5^ CD19^+^ B cells is reported.

On the contrary, the analysis of circulating B cells from patient 9 with CD19, CD27, and IgD markers indicated a frequency of mature (CD27^+^) and switched (CD19^+^/IgD^−^/CD27^+^) B cells similar to that observed in age-matched healthy control (Fig. 4*A*). Examination of B cell development in the bone marrow revealed a reduced frequency of early-immature B cell subsets but rather normal to increased percentages of naïve and memory B cells with respect to the control (Fig. 4*B*). Moreover, ELIspot analysis showed, in patient 9 and in the healthy donor, similar frequencies of immunoglobulin-secreting cells in the peripheral blood and bone marrow, either producing IgM or IgG/A (Fig. 4*C*). Consistent with these findings, the patient's global Ig levels were in the normal range (Table 1), as well as specific antibody titers against live vaccination.

### HSCT and follow-up

All the patients received HSCT. Myeloablative conditioning, prevention of veno-occlusive disease (VOD) and prophylaxis of graft-versus-host disease (GVHD) were performed according to the European Group for Bone Marrow Transplantation–European Society for Immunodeficiencies (EBMT-ESID) guidelines.

Patient 8A was transplanted at 21 months of age with peripheral stem cells from a matched unrelated donor (10/10) without T cell depletion. For prophylaxis of GVHD Cyclosporin A was replaced by corticosteroids at day +16 because of important hypercalcemia. Myeloid engraftment occurred on day +15, platelet counts and red blood cell counts rapidly normalized (last transfusion on day +10 and on day +29, respectively). Full donor engraftment was achieved. Severe hypercalcemia (plasma calcium level up to 4.02 mmol/L and ionized calcium 2.13 mmol/L at day +18) required hyperhydration, diuretics, corticosteroids, bisphosphonates, and calcitonin for several weeks. Two months after HSCT the patient developed nephrocalcinosis; however, renal function could be completely restored. Besides hypercalcemia, the post-HSCT course was uneventful and favorable. The patient is alive and well, without any specific treatment, at the last follow-up 18 months after HSCT. He presents no developmental delay in his motor milestones and his mental development is very satisfying with regard to language and understanding; however, no obvious improvement in visual ability occurred. The first teeth were delayed in their eruption: the patient had 11 teeth at the age of 33 months.

Patient 8B received an HLA-identical family donor HSCT at 5 months of age. Myeloid engraftment occurred on day +21, platelet counts and red blood cell counts rapidly normalized. At engraftment, donor chimerism was 95%. Ionized calcium was at maximum 1.48 mmol/L at day +20, hypercalcemia was rapidly regressing under hyperhydration. At day +8, the patient presented very mild signs of VOD, evolution was rapidly favorable. At day +46, the patient had to be transferred to the pediatric intensive care unit for acute respiratory failure because of *Pneumocystis* pneumonia and suprainfection with *Klebsiella pneumoniae*. The course was favorable under adequate antiinfectious therapy. The patient was dismissed 3 months after HSCT and is alive and well at the last follow-up 6 months after HSCT.

Patient 9 received a matched family donor stem cell transplantation at 12 years of age. Full donor engraftment was associated with severe hypercalcemia (plasma calcium level up to 4.2 mmol/L) requiring hemodialysis, bisphosphonates, calcitonin, and finally anti-RANKL antibody, which resulted in normalization of plasma calcium levels (manuscript in preparation). About 12 months after transplantation she was discharged from hospital. About 15 months after transplantation, she is in very good mental condition, her blood cell counts and her serum calcium levels have normalized. The tracheostomy is still in place and immunosuppressive treatment for limited chronic GVHD and antimicrobial treatment because of osteomyelitis of the jaw are ongoing.

Patient 10 received a matched unrelated donor (10/10) stem cell transplant at the age of 3 years, achieving full donor engraftment. Six weeks after transplantation she developed cyclosporine-induced posterior reversible encephalopathy (PRES), so calcineurin inhibitor was stopped and replaced with Sirolimus and steroids to prevent GVHD. Two months after transplantation she presented persistent hypercalcemia (maximum level 5 mmol/L) treated unsuccessfully with hemofiltration. Plasma calcium level normalized after receiving anti-RANKL antibody (manuscript in preparation). The patient also developed respiratory distress and oxygen desaturation because of a combination of upper respiratory obstruction and aspiration pneumonia. A tracheostomy was performed and the patient is still on continuous positive airway pressure (CPAP) support, 4 months after transplantation.

Patient 11 underwent a matched sibling donor bone marrow transplantation at the age of 11 months. The transplant procedure and posttransplant recovery period was uncomplicated, with only mild hypercalcemia (maximum level 3.39 mmol/L) not requiring specific intervention. The child achieved full donor engraftment and a good immune reconstitution. Eight months post-HSCT he developed diarrhoea for which he had biopsies, suggesting an eosinophilic enterocolitis treated with Cetirizine and Sodium Cromoglycate. Because of a lack of interest in feeding the patient eventually had a gastrostomy inserted at about 10 months post-HSCT, with subsequent weight gain. He had a skeletal survey 19 months post-HSCT and, compared with a pre-HSCT study, bones showed satisfactory improvement in their structural appearances. He is now 3 years post-HSCT, at home and reasonably well. He has a mild developmental delay especially in his motor milestones; his sleep is disturbed and unpredictable.

## Discussion

Autosomal recessive osteopetrosis is a rare genetic bone disease in which the main feature, increased bone density, is due to either the presence of nonfunctional osteoclasts (in the osteoclast-rich forms) or to their absence (in the osteoclast-poor forms). In humans these latter represent a very limited group which has been identified only very recently.^3^, ^4^, ^13^, ^14^ At least two genes, *TNFSF11* and *TNFRSF11A,* are involved in their pathogenesis. In the present work we report the clinical and molecular characterization of five new patients affected by osteoclast-poor ARO, because of novel mutations in the *TNFRSF11A* gene. In our cohort of more than 250 ARO, RANK-dependent patients constitute almost 5%, and to our knowledge, no other cases of this subgroup have been described. Overall, the clinical phenotype of the five new patients closely resembled that of the RANK-dependent ARO we have previously described; they presented with a classical osteopetrotic bone appearance and a variability of fracture incidence; blindness was present in four out of five patients and other secondary neurological defects were reported in two. On the basis of the phenotype of *Tnfrsf11a*^−/−^ mice and of our previous preliminary observation suggesting a primary immunological defect in RANK-dependent ARO, we performed immunological investigations in three patients, mainly focused on the B cell compartment.

Our results suggest that the degree of immunological impairment of RANK-ARO patients is variable. In patient 9 a low number of B cells was present in the periphery compared with the corresponding healthy donor; however, we could not find a major defect in B cell maturation as confirmed by normal Ig levels. On the contrary, patients 8A and 8B showed a reduction in switched memory B cells, even though less pronounced than in patients 1A and 1B from our previous work, suggesting that the defect could worsen with age or the progression of the disease. The variability in Ig levels could be explained in the same way, even though further investigation is required to clarify this aspect. Interestingly, low IgG levels have also been recently reported in a few TCIRG1-dependent ARO.^15^

All five patients were transplanted and in three of them the bone defect was rescued, while bone remodelling is still ongoing in patients 8B and 10. The favorable outcome of HSCT further confirms the appropriateness of this therapeutic approach in RANK-dependent ARO, but as expected, it cannot restore the visual capacity if the optical nerve is already damaged at the time of transplantation. This fact emphasizes the essential role of molecular diagnosis and highlights that RANK-patients should be transplanted as early as possible, despite the absence of severe hematological features, in order to prevent visual loss.

Of note, patient 9 is, to our knowledge, the only ARO patient transplanted late in childhood (12 years compared with a mean age of 10.3 months and 6 months in 2 different reports);^15^, ^16^ however, HSCT was successful with persistent severe hypercalcemia as the only side effect. Hypercalcemia is a well-known post-HSCT complication in ARO,^16^–^19^ but in the RANK-dependent patients here reported, it seems to have a higher prevalence and a longer persistence, requiring more aggressive treatments. A correlation was suggested between hypercalcemia and age at HSCT,^16^, ^19^ leading to the hypothesis that larger bone mass in older patients could result in a higher and longer release of calcium stored in the osteopetrotic bones by donor-derived osteoclasts activity. Indeed, RANK-dependent patients received HSCT later in life, owing to a milder hematological involvement, compared with classic TCIRG1-dependent osteopetrosis. Another explanation might be that in RANK-dependent patients, *TNFSF11* may be compensatorily overexpressed, leading to a highly activated RANKL-RANK system which, after transplant, may generate a rapid production and an overactivity of osteoclasts. The fact that anti-RANKL antibody worked effectively and quickly in patients 9 and 10 would support this latter hypothesis; however, RANKL serum levels were not assessed and these two hypotheses are not mutually exclusive.

The molecular analysis of these five new patients led to the identification of five novel mutations in the *TNFRSF11A* gene: two missense mutations (p.Ala134Val and p.Asp148Val), two nonsense (p.Glu83X and p.Cys124X), and a single-nucleotide insertion leading to frameshift and premature termination (p.Arg110ProfsX52). They are all located in the extracellular region of the protein, in particular, p.Ala134Val and p.Asp148Val are in the fourth cysteine rich domain (CRD4) which, according to the recent crystallographic model of the murine protein,^20^, ^21^ is not directly involved in the binding of the ligand. However, the mutated residues appeared to be strongly conserved in evolution, so it could be suggested that amino acid substitutions at these positions might alter the folding of the ectodomain and, as a consequence, the interaction with RANKL. A similar hypothesis has been raised for three mutations we reported in our previous work,^4^ namely, p.Gly53Arg, p.Arg170Gly and p.Cys175Arg.^20^, ^21^

The two nonsense mutations and the insertion are predicted to lead to the production of truncated forms of RANK protein, lacking the entire transmembrane and intracellular domains. As hypothesized by Crockett and colleagues^5^ for other truncating mutations previously identified in *TNFRSF11A*, the nonsense-mediated mRNA decay process is likely to lead to the destruction of a significant proportion of the mRNA molecules transcribed from these mutated alleles resulting in reduced levels of *TNFRSF11A* expression. However, for these novel mutations in the N-terminal domain, the possibility of reinitiation from a downstream methionine codon cannot be excluded, as has previously been reported in several diseases.^22^–^24^ The putative shorter transcript could maintain a partial activity and be responsible for the milder phenotype of patient 9, who is homozygous for the mutation p.Arg110ProfsX52. However, the analysis at the protein level was not possible either in this patient or in patient 11, who is compound heterozygous for p.Glu83X and p.Cys124X mutations. In addition preliminary in vitro experiments using expression constructs bearing the mutant sequences were inconclusive (data not shown); therefore, this possibility remains completely speculative.

In conclusion, RANK-dependent ARO is confirmed to benefit from HSCT, although patients seem to be particularly prone to hypercalcemia in the post-HSCT period, especially when HSCT is carried out at an older age. Defects of the humoral immune system can be present, but apparently not as severe as in the corresponding knockout murine model. These data add to the clinical and molecular heterogeneity of human ARO and further confirm the important role of a precise molecular diagnosis with respect to therapy.

## Supplementary material

Supplementary Figure 2. (A) Highly mineralised bone tissue revealed multiple cutting artifacts (red arrow) and irregular net-like trabecular structure (white arrow). Intertrabecular spaces (yellow arrow) were scarce and showed neither adipocytes nor islands of haematopoiesis (embedding in methyl-methacrylate without decalcification, stain: toluidine blue, original magnification: x50). (B) Detail. Irregular bone trabeculae contained preferentially a mineralised primary spongiosa similar to calcified cartilage (white arrow). Lamellar structured mature bone tissue was not present. However, few osteocytes surrounded by a ring of more mature appearing bone were apparent both within the primary spongiosa (red arrow) and on the trabecular surface. Although few flat (lining cell-like) cells were evident on the trabecular surface, no sign of metabolic active osteoblasts was found in the biopsy specimen. Osteoclasts were not detected using conventional staining methods. Vascularised loose fibrotic tissue with scattered isolated haematopoietic cells occupied the intertrabecular spaces (embedding in methylmethacrylate without decalcification, stain: Giemsa, original magnification: x400).
