# Supplementary figures and images for: RANK-Dependent Autosomal Recessive Osteopetrosis: Characterization of Five New Cases With Novel Mutations
Source: J Bone Miner Res. 2011 Nov 9;27(2):342–51. doi: 10.1002/jbmr.559 (PMC3306792; doi:10.1002/jbmr.559)

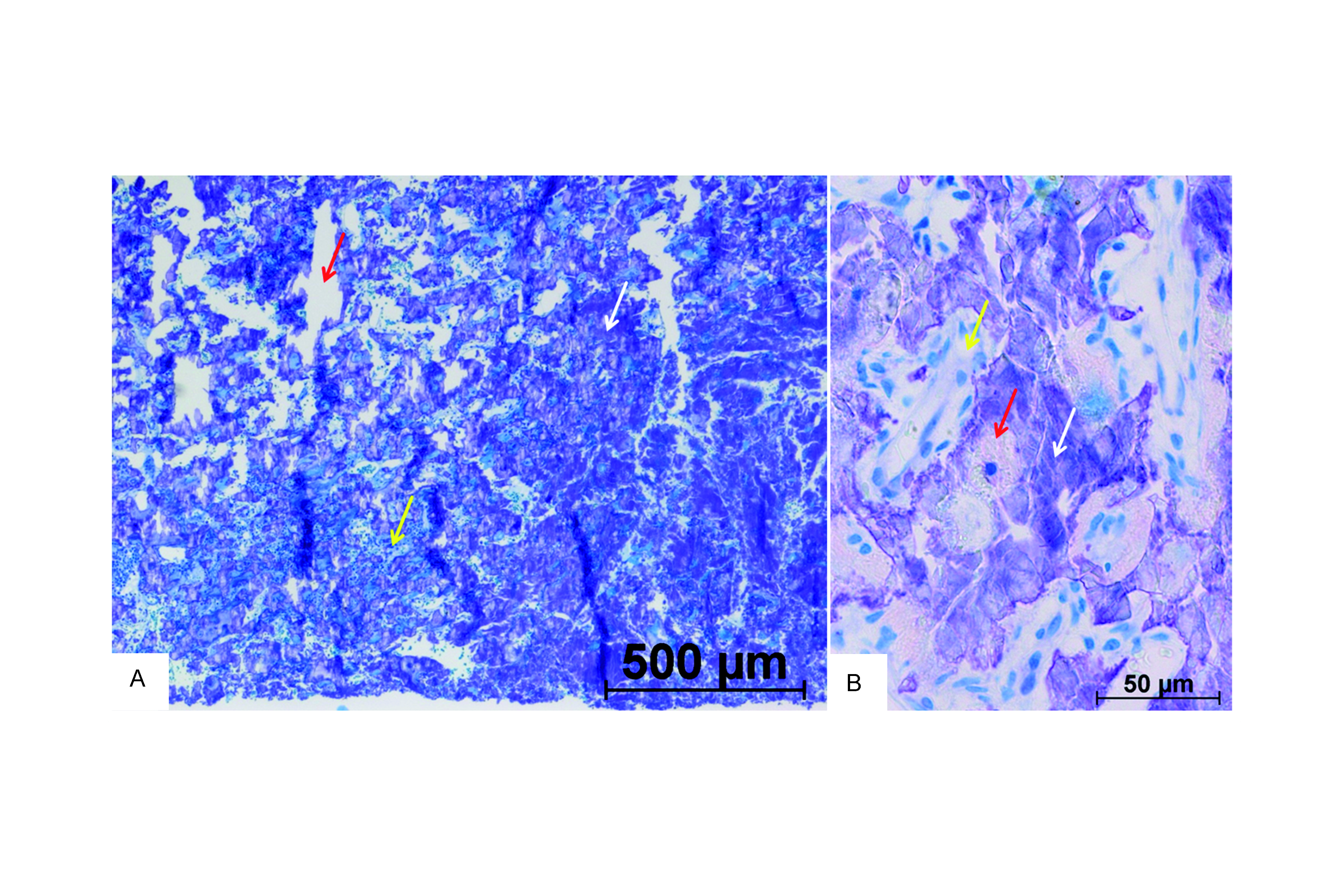

Supplement: Supplementary file 2 [file jbmr0027-0342-SD2.tif]
